# Nanotechnology-based formulations of thyme essential oil to enhance its herbicidal efficiency for controlling weeds associated with maize plants

**DOI:** 10.1186/s12870-026-08928-6

**Published:** 2026-05-25

**Authors:** Faten S.A. Zaki, Mona A. El-Wakeel, Alaa E. El-Sayed

**Affiliations:** 1 Botany Department, Agriculture and Biological Institute, National Research Centre, Giza, Egypt; 2 Polymers and Pigments Department, Chemical Industries Research Institute, National Research Centre, Giza, Egypt

**Keywords:** Weed control, Maize, Thyme essential oil, Nanoemulsion, Nano-encapsulation, β-cyclodextrin

## Abstract

**Background:**

Thyme is an aromatic plant known as a rich source of bioactive compounds with eco-friendly herbicidal potential. Despite the herbicidal potential of thyme essential oil, its effectiveness under field conditions is limited due to its high volatility, poor water solubility and susceptibility to environmental degradation. Nanoformulations can overcome these limitations by enhancing stability, dispersibility, bioavailability, and prolonging herbicidal activity. Hence, this study was applied to evaluate and compare the effectiveness of aqueous extracts from thyme leaves and thyme essential oil against weeds. A preliminary pot experiment tested different concentrations of thyme leaf aqueous extracts (0, 10, 20 and 30% w/v) and thyme essential oil (0, 3, 6 and 9% v/v). Based on these results, two subsequent pot experiments were conducted during two consecutive summer seasons using lower concentrations (0, 2, 4 and 6% v/v) of crude thyme essential oil, its nanoemulsion and β-cyclodextrin nanoencapsulated formulations. Healthy weed-free maize plants and unweeded controls were included for comparison.

**Results:**

The preliminary experiment revealed that thyme essential oil was more efficient in controlling weeds than aqueous extracts in weed control, where 9% thyme essential oil achieved complete suppression with stimulatory response in maize plants (11.4 g dry biomass) but this stimulatory response was lower than 6 and 3% concentrations (11.9 and 11.5 dry biomass, respectively). In the main experiment, nanoemulsion and β-cyclodextrin significantly outperformed raw essential oil in weed suppression and crop safety. At 90 DAS, NE-Th oil at 6 and 4% achieved the highest reduction in barnyard grass dry weight (83.48 and 81.72%, respectively) and purslane (89.27 and 82.41%, respectively) compared to the unweeded control. Regarding maize plants at 90 DAS, NE-Th oil at 6 and 4% showed the best performance, significantly exceeding the unweeded control dry weight (50.83 and 45.48 g), respectively. These results indicate that nanoformulations not only enhance weed suppression but also improve crop performance under weed stress competition.

**Conclusion:**

Both nanoemulsion and β-cyclodextrin inclusion complex formulations enhanced the stability and herbicidal efficiency of thyme oil. Therefore, nanoemulsified thyme oil at 4% and 6% achieved the best balance between effective weed suppression and improved maize growth.

**Graphical Abstract:**

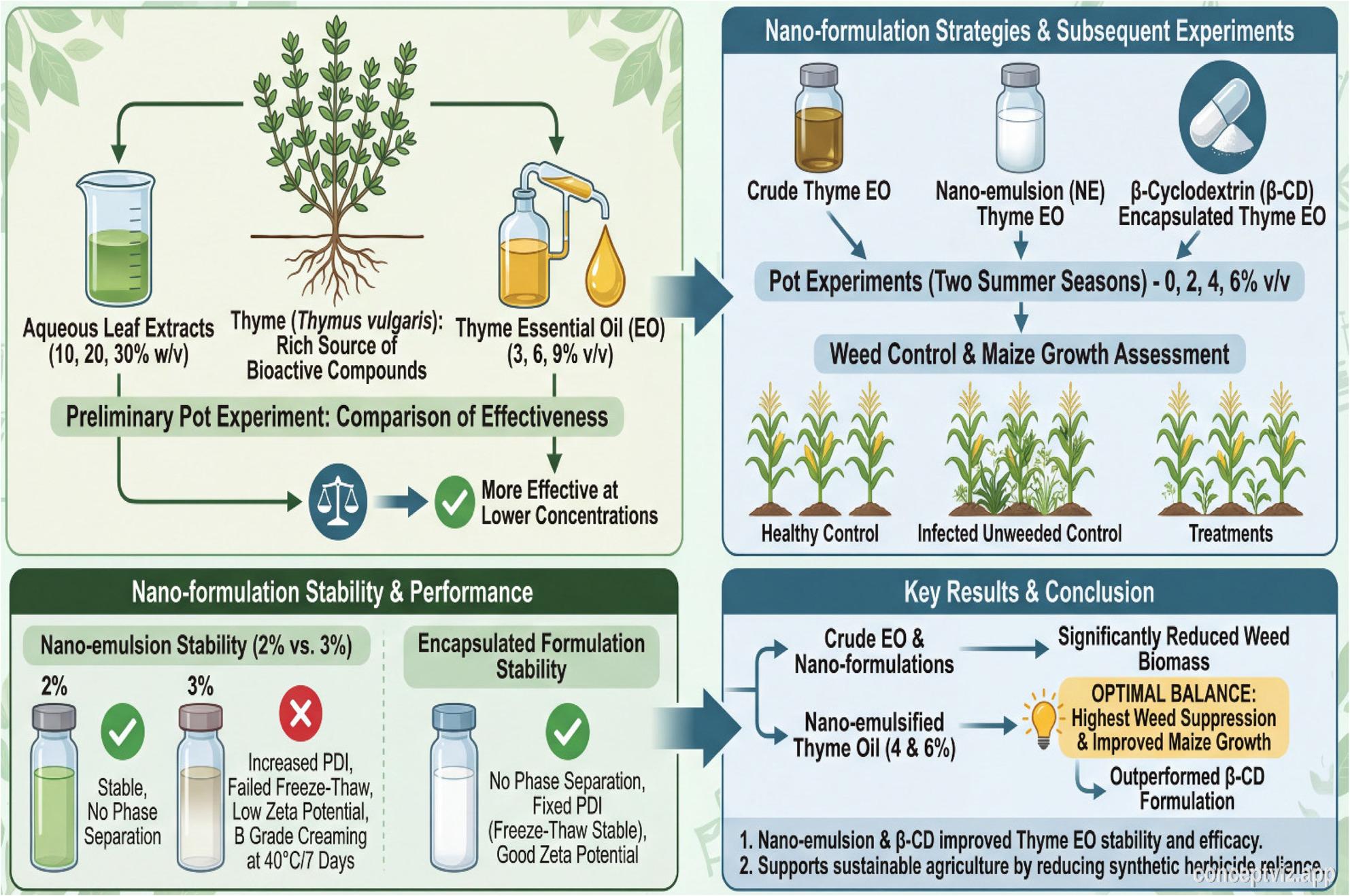

## Introduction

Maize (*Zea mays* L.) is among the most globally significant cereal crops, serving as a major food source and an essential component of animal feed systems for more than one billion people worldwide [1]. Global demand for maize is projected to exceed current production levels, with an estimated annual increase of about 37 million tons by 2050, mainly driven by population growth, dietary changes, and the rising need for livestock feed [2]. Consequently, overcoming constraints to maize production remains a key priority for achieving food security and supporting economic development on a global scale [3]. Weed competition remains a major constraint in agricultural systems through reducing crop yield and quality while increasing the need for repeated herbicide applications. Although synthetic herbicides are effective, their widespread use has been associated with environmental pollution, non-target toxicity, and the evolution of herbicide-resistant weeds, prompting a shift toward natural and sustainable weed management solutions [4].

Essential oils (EOs) from aromatic plants have gained attention as bioherbicides due to their complex mixtures of aromatic secondary metabolites that can inhibit seed germination and seedling growth of weed species [5]. *Thymus* vulgaris (thyme) belongs to Lamiaceae family, has been particularly proven to be a promising bioherbicide because of high levels of terpenoid allelochemicals like thymol, carvacrol, and p-cymene that exhibited significant herbicidal potential against *Lolium perenne* L. and *Amaranthus retroflexus* L [6]. EOs from thyme also have demonstrated significant phytotoxic effects on weeds, including *Chenopodium album* and *Echinochloa crus-galli*, supporting their potential use as natural herbicide agents [7]. Additionally, recent work has evaluated the herbicidal effects of Thyme EOs, reporting that Thyme EOs can strongly reduce weed seedling growth of *Avena fatua* L. and *Malva parviflora* L. under greenhouse conditions [8].

Despite the ecofriendly promise of thyme EOs, its practical application in field settings is limited by its volatility, poor water solubility and susceptibility to environmental degradation, which reduce persistence and efficacy when applied directly as a natural herbicide due to exposure to light and oxygen during storage or application [9]. To overcome these challenges, nanotechnology-based formulations, particularly nanoemulsions (NEs), have been investigated. By encapsulating essential oils (EOS) in nanoscaled carriers, these delivery systems can enhance stability, water dispersibility, and bioavailability, potentially improving contact with plant tissues and prolonging activity [10]. Although much of the work on EOs nanoemulsions has focused on antimicrobial or insecticidal applications, similar principles apply to herbicidal use. For instance, EOs nanoemulsions have shown improved biological activity and stability compared with unformulated oils in related agricultural contexts [11, 12]. These features contribute to reduced volatilization and slower degradation of active compounds, thereby promoting greater penetration into target tissues and enhanced biological activity [13]. Moreover, NE formulations are particularly important for dispersing lipophilic or poorly water-soluble substances, such as EOs, agrochemicals, and food additives, in aqueous systems [14].

Beyond nanoemulsification, β-cyclodextrin (β-CD) represents a complementary encapsulation strategy with direct relevance to herbicidal applications. β-CD possesses a hydrophobic internal cavity capable of hosting lipophilic monoterpenoids, the primary bioactive constituents of thyme EO such as thymol and carvacrol thereby protecting them from volatilization and oxidative degradation during storage and field application [15, 16]. This encapsulation converts the liquid EO into a water-dispersible solid powder, facilitating uniform application and controlled release of phytotoxic volatiles upon contact with the soil or plant surface [17, 18]. β-CD encapsulation preserves and enhances the herbicidal activity of thyme oil, with complexes of carvacrol and thymol showing greater phytotoxic potency than the free compounds, supporting more effective and sustained weed control [19, 20]. Furthermore, the chemical compatibility between the terpenoid compounds and the β-CD cavity determined how efficiently the oil can be encapsulated and released, which in turn controls the duration and strength of its herbicidal activity Applied to weed management. Hence, β-CD–thyme oil complexes can extend the window of herbicidal action and reduce the required dose frequency, supporting sustainable, low-input agricultural practices [20].

Nevertheless, relatively few studies have addressed how the encapsulation technique influences the stability of EOs with different chemical profiles. Barbieri et al. [21] investigated the encapsulation of various *Lippia graveolens* chemotypes in β- and γ-cyclodextrins (β-CD and γ-CD). Their findings indicated that oils with higher concentrations of carvacrol and thymol exhibited stronger affinity toward γ-cyclodextrin, whereas oils rich in β-caryophyllene achieved greater encapsulation efficiency in β-cyclodextrin. However, after 14 days of storage, carvacrol levels remained stable in the β-CD complexes, while a significant reduction was observed in the γ-CD complexes. These results suggest that the interaction between EO constituents and the encapsulating matrix plays a decisive role in determining the stability of the encapsulated system.

Although recent studies revealed that thyme essential oil has shown bioherbicidal potential against various weed species but most studies have tested it only in its crude, unformulated form, leaving critical gaps in translating this efficacy into practical agricultural applications [6–8]. Such approaches do not reflect the physicochemical challenges encountered during actual foliar application in the field, where rapid volatilization, photodegradation, and poor aqueous dispersibility collectively reduce the persistence and bioavailability of active terpenoid constituents on target weed tissues [9]. Critically, no study to date has directly compared the herbicidal performance of crude thyme EO, its nanoemulsion formulation, and its β-cyclodextrin inclusion complex within the same experimental framework, nor evaluated their simultaneous effects on both weed suppression and crop growth responses across multiple growth stages. This represents a fundamental gap in the evidence base needed to guide the rational development of thyme EO-based bioherbicide formulations for integrated weed management in cereal crops such as maize.

Hence, this study compared three different application strategies of thyme essential oil (EO) in a greenhouse pot experiment: unformulated crude oil, nanoemulsion (NE-Th oil), and β-cyclodextrin nanoencapsulation (β-CD-Th oil) at various concentrations and growth stages. The study is novel in three ways. Firstly, it directly compared nanoemulsion and β-cyclodextrin encapsulation as delivery systems for thyme EO, providing insights into which strategy is most effective against economically important weed species associated with maize production. Secondly, it went beyond previous studies by not only assessing the herbicidal effects on weed biomass but also monitoring maize responses, including growth parameters, photosynthetic pigment content, total soluble sugars, and total phenolic compounds in leaves, in order to establish the selectivity window of each formulation. Thirdly, the study linked the physicochemical characterization of the nanoformulations to their observed biological performance, providing a formulation–efficacy relationship largely absent from existing bioherbicide literature. Overall, the study focused on identifying the most efficient formulation in terms of weed suppression, stability, and bioactivity, thereby offering insights into the optimal utilization of thyme oil as a natural herbicide within integrated weed management programs.

## Materials and methods

### Chemicals and investigated material

All chemicals and reagents utilized in this research were of analytical grade and acquired from reputable vendors. β-cyclodextrin (β-CD) was obtained from Sigma-Aldrich, Germany. The plant material (*Thymus vulgaris* L.) was purchased from a reliable local market as whole plants and taxonomically identified by a specialist from the Department of Pharmacognosy, Faculty of Pharmacy, Badr University in Cairo (BUC), Egypt. A voucher specimen was deposited in the herbarium of the same department under voucher codes BUC-PHG-TV-31 (*Thymus vulgaris*). Maize (*Zea mays*) (cultivar Three Way Cross–310), barnyard grass (*Echinochloa crus-galli*) and purslane (*Portulaca oleracea*) seeds were obtained from the Agricultural Research Centre, Giza, Egypt.

#### Essential Oils (EOs) extraction

According to Kamyab et al. [22], essential oils were extracted from 5 kg dried aerial parts of thyme plants using a Clevenger-type apparatus and a 3-hour hydro-distillation process. After being extracted, the oils were dried over anhydrous sodium sulfate (Na_2_SO_4_) and kept between 4 °C until further examination. Accordingly, the essential oil content was calculated as a relative percentage (v/w) 0.95%. The extraction process was repeated as needed to obtain the required amount of essential oil, ensuring that the oil remained in good quality.

#### GC-MS fractionation of thyme oil terpenoids

The sample was dissolved in dichloromethane and injected into Gas chromatography–mass spectrometry (GC–MS). GC-MS analysis was conducted using an Agilent 7890B gas chromatograph coupled to a 5977 A mass selective detector (Agilent Technologies). Separation was achieved on an HP-5MS capillary column (15 m × 0.25 mm i.d., 0.25 μm film thickness) with hydrogen as the carrier gas at 1.1 mL/min. Samples (1.0 µL) were injected in splitless mode. The oven temperature was programmed from 40 °C (1 min) to 200 °C at 10 °C/min (1 min hold), then to 220 °C at 20 °C/min (1 min hold), and finally to 300 °C at 30 °C/min with a 3 min hold. Injector and detector temperatures were maintained at 250 °C and 300 °C, respectively. Mass spectra were recorded under electron ionization (EI) at 70 eV over an m/z range of 33–600, with a solvent delay of 2.0 min. The mass temperature was 230 °C and Quad 150 °C. Identification of different constituents was determined by comparing the spectrum fragmentation pattern with those stored in Wiley and NIST Mass Spectral Library data [23].

#### Preparation of thyme leaves aqueous extract

Thyme leaves aqueous extract stock solution (50% w/v) was prepared for investigation as described by Andualem et al. [24], 500 g of thyme leaf powder were placed in a 2 L Erlenmeyer flask and 1 L distilled water was added. The mixture was shaken at 200 rpm at room temperature for 48 h. To remove debris, the mixture was filtered through four layers of cheesecloth and centrifuged at 4000 rpm for 30 min. After that, the supernatant was filtered through one layer of filter paper (Whatman No. 1). Using distilled water, three concentrations of 10, 20 and 30% (w/v) were prepared from a 50% stock solution. The extraction method was repeated as needed to ensure that the extracts were always fresh.

#### Preparation of oil extract

Thyme essential oil extract in aqueous phase was prepared by adding 1 mL of Tween 20 to each prepared concentration (3, 6, and 9% v/v thyme essential oil), and the total volume of each formulation was adjusted to 300 mL with distilled water. The mixture was thoroughly homogenized to obtain a uniform dispersion.

#### Preparation of thyme oil nanoformulations

##### Nanoemulsion Thyme oil (NE-Th oil)

The nanoemulsion of thyme essential oil (NE-Th oil) was prepared using a combined surfactant system consisting of Span 80 (lipophilic, HLB ~ 4.3) as the primary emulsifier and Tween 80 (hydrophilic, HLB ~ 15) as a co-surfactant, selected to achieve a balanced interfacial film at the oil-water interface. The oil phase was prepared by dissolving Span 80 (2% w/w) directly into thyme essential oil (2% w/w) at 50 °C with gentle stirring until complete dissolution was achieved. The aqueous phase was prepared separately by dissolving Tween 80 in distilled water (96% w/w total) at the same temperature. The oil phase was then added dropwise into the aqueous phase under continuous high-shear homogenization at 15,000 rpm for 20 min, resulting in the formation of a fine oil-in-water (O/W) nanoemulsion. The total Span 80 and thyme nanoemulsion oil content in the final formulation ranged from 1 to 3% and 1–3% (w/w), respectively, depending on the treatment concentration. All nanoemulsion batches were freshly prepared prior to application to ensure physicochemical stability and consistent herbicidal bioactivity [13, 25].

##### β-cyclodextrin thyme oil nanoencapsulation (β-CD-Th oil)

The inclusion complex β-CD-Th was prepared by co-precipitation according to Ozogul et al. [26]. β-CD (10 g) was solubilized in ethanol solution (10% v/v) and heated to 55 °C. Later, different amounts of thyme oil were added: 2.0, 4.0 and 6.0% (w/w), respectively. The solution was stirred for 4 h at room temperature and stored at 4 °C in the refrigerator overnight. Filtration of the solution was carried out under vacuum, followed by drying for 24 h at 55 °C. The dried inclusion complex (IC) powders were stored for a maximum of 24 h until the analyses were carried out.

#### Characterization of formulated nanoemulsions

##### Foam test

The physical characteristics of the thyme oil nanoemulsions were evaluated through droplet size analysis and foam stability tests. The mean droplet size and polydispersity index (PDI) were determined using Dynamic Light Scattering (DLS) to ensure the formulation maintained a nanoscale distribution (below 200 nm), which is critical for leaf cuticle penetration. Furthermore, foam behavior was assessed by shaking 50 mL of the emulsion in a 250 mL graduated cylinder (amplitude 30–40 cm). The initial foam height (H_0_​) was recorded after 1 min, and stability was tracked by height reduction over 30 min. For emulsification, a surfactant blend of 1.5–2.5% Span 80 and 0.5% Tween 80 was utilized to achieve an optimal Hydrophilic-Lipophilic Balance (HLB). All measurements were conducted in triplicate (*n* = 3) to ensure the reliability of the results [27, 28].

$$\mathrm{Measurement:}\;\mathrm{H}_{0}\;\text{after 1 min,}\;\mathrm{H}_{30}\;\text{after 30 min;}\;\mathrm{stability}=\left(\mathrm{H}_{30}/\mathrm{H}_{0}\right)\times100$$

##### Stability test

Formulations were evaluated using several accelerated stress tests. The formulations were subjected to six heating–cooling cycles between 4 and 45 °C (48 h each) and three freeze–thaw cycles between − 21 and 25 °C (48 h each). Centrifugation tests were also performed at 3,500–10,000 rpm for 30 min to detect possible phase separation, creaming, or cracking. Droplet size and polydispersity index (PDI) were measured using dynamic light scattering (DLS) before and after the stress tests. Zeta potential was also determined to evaluate the electrostatic stability of the formulations. In addition, visual observations were conducted to monitor turbidity changes and creaming behavior, which were qualitatively assessed using a grading scale (Grades A–D) according to the visual appearance of the emulsion [29].

##### Particle size and zeta potential

At room temperature, the Zetasizer Nano ZS (Malvern Instruments, UK) was used to measure the size of the nanoemulsion droplets using DLS. Prior to testing, 30 µl of the nanoemulsion was diluted with 3000 µl at 25 °C. Particle size information was expressed using the mean of the Z-average of three distinct batches of the nanoemulsion. The zeta potential of the nanoparticles was measured at 25 °C to estimate the surface charge of each formulation [30].

#### Experimental design and treatments

##### Preliminary pot experiment

A preliminary exploratory pot experiment was conducted in the first week of June during the summer season of 2023 to evaluate and compare the efficacy of aqueous and oil extracts derived from thyme leaves. The experiment was applied at the greenhouse of the National Research Centre, Dokki, Giza, Egypt, under a random complete block design with three replicates (RCBD). All pots (20 cm diameter) were filled with clay-loam soil (pH 7.8, EC 1.2 dS/m). All pots were sown with sown with 3 maize seeds, barnyard grass seeds and purslane seeds except the pots of healthy weed-free maize plants. After 10 days maize plants were thinned to one plant/pot. Eight treatments were applied as follows: three treatments contained three concentrations for each thyme aqueous extract (10, 20 and 30% w/v), three thyme oil extracts (3, 6 and 9% v/v), and two control treatments of healthy weed-free maize plants (plant only) and unweeded control (maize plants and both weeds). Both prepared extracts were sprayed via spraying at 21 and 30 days after sowing (DAS), with each extract applied twice using a hand sprayer at a rate of 50 mL per pot. Additionally, distilled water was utilized to implement two control treatments: healthy plants and mixed control for comparative analysis. The eight experimental treatments are detailed in Table 1. All treatments were maintained under greenhouse controlled conditions with a temperature range of 28 ± 2∘C during the day and 22 ± 2∘C at night. The relative humidity was maintained at 60–65%, under a natural photoperiod (approx. 14 h light). and standard cultural practices were followed; particularly concerning fertilization and irrigation regime was maintained at 70% of field capacity to avoid water stress.

**Table 1:** Description of experimental treatments

Treatment No.	Treatment Name	Concentration	Details
T1	Weed-free Control	0%	Maize only + Distilled water spray
T2	Weed-infested (unweeded ) Control	0%	Maize + Weeds + Distilled water spray
T3	Thyme Aqueous Extract	10% (w/v)	Foliar spray at 21 & 30 DAS
T4	Thyme Aqueous Extract	20% (w/v)	Foliar spray at 21 & 30 DAS
T5	Thyme Aqueous Extract	30% (w/v)	Foliar spray at 21 & 30 DAS
T6	Thyme Oil Extract	3% (v/v)	Foliar spray at 21 & 30 DAS
T7	Thyme Oil Extract	6% (v/v)	Foliar spray at 21 & 30 DAS
T8	Thyme Oil Extract	9% (v/v)	Foliar spray at 21 & 30 DAS

##### Main pot experiments

Two pot experiments were carried out in the second week of June during two successive summer seasons of 2024 and 2025 in the greenhouse of the National Research Centre, Dokki, Giza, Egypt. The greenhouse was maintained under conditions reflecting the natural ambient environment: Temperature: Average day/night temperatures of 25 ± 2 °C / 15 ± 2 °C. Relative Humidity: 60%±5%. Light Intensity: 800–1000µmol/m²/s (PAR) during peak daylight. Photoperiod: Natural light (approximately 11–12 h). All pots (30 cm in diameter and 35 cm in depth) were filled with 10 kg of filled with clay-loam soil. Soil analysis revealed a pH of 7.65, organic matter of 1.85%, and available N, P, and K at 45.2, 12.8, and 180.5 mg/kg, respectively. All pots were sown with 3 maize seeds and 10 wild barnyard grass seeds as well as 10 purslane seeds at 5 cm depth from the soil surface except the healthy weed-free control. The sown seeds were arranged at equal distances to ensure uniform distribution. After 10 days maize seedlings were thinned to 2 seedlings in all pots. As listed in Table 2, Eleven treatments and six independent replicates, where each individual pot served as the experimental unit in a complete randomized block design (RCBD) as follows: Nine oil treatments contained three phases of thyme oil i.e. pure thyme oil, nanoemulsion thyme oil (NE-Th oil) and β-cyclodextrin thyme oil nanoencapsulation (β-CD-Th oil) at three rates of concentrations (2, 4 and 6%) v/v and two control treatments of healthy weed-free maize plants (plant only) and unweeded infested control (maize plants and both weeds). The prepared oil extracts were sprayed via spraying at 21 and 30 DAS, each extract applied twice using a hand sprayer at a rate of 50 mL per pot. Additionally, distilled water was utilized to implement two control treatments healthy plants and mixed control for comparative analysis. All treatments were maintained under greenhouse conditions and standard cultural practices were followed, particularly concerning fertilization and irrigation.

**Table 2:** Description of experimental treatments

Treatment No.	Treatment Name	Concentration	Details
T1	Weed-free Control	0%	Maize only + Distilled water spray
T2	Weed-infested (unweeded ) Control	0%	Maize + Weeds + Distilled water spray
T3	Thyme oil	2% (w/v)	Foliar spray at 21 & 30 DAS
T4	Thyme oil	4% (w/v)	Foliar spray at 21 & 30 DAS
T5	Thyme oil	6% (w/v)	Foliar spray at 21 & 30 DAS
T6	NE-Th oil	2% (v/v)	Foliar spray at 21 & 30 DAS
T7	NE-Th oil	4% (v/v)	Foliar spray at 21 & 30 DAS
T8	NE-Th oil	6% (v/v)	Foliar spray at 21 & 30 DAS
T9	β-CD-Th oil	2% (v/v)	Foliar spray at 21 & 30 DAS
T10	β-CD-Th oil	4% (v/v)	Foliar spray at 21 & 30 DAS
T11	β-CD-Th oil	6% (v/v)	Foliar spray at 21 & 30 DAS

#### Sampling and measurements

##### Preliminary pot experiment

**Weeds**

Three replicates (pots) were collected from each treatment at 45 days after sowing (DAS). The fresh weight of each weed (g/pot) was recorded. Weed samples were classified into grass weeds of barnyard grass and broadleaf weeds of purslane. The freshly harvested weed samples were initially air-dried for 10 days, followed by oven drying at 80 °C for 72 h until a constant weight was achieved. The individual weed dry biomass was then measured and expressed as dry weight per pot.

**Maize plants**

*Growth traits*

In both seasons, samples of maize plants at 45 DAS were collected from each treatment to determine shoot height (cm), number of leaves/ plant, fresh weight (FW) of plant (g), dry weight (DW) of plant (g).

##### Main pot experiments

**Weeds**

Three replicates (pots) were collected from each treatment at 45 days after sowing (vegetative growth age), another three replicates were collected 90 DAS (late of flowering growth age). The fresh weight of each weed (g/pot) was recorded at the two growth ages. Weed samples were classified into grass weeds, barnyard grass and broadleaf weeds of purslane. The freshly harvested weed samples were initially air-dried for 10 days, followed by oven drying at 80 °C for 72 h until a constant weight was achieved. The individual and total weed dry biomass was then calculated and expressed as dry weight per pot.

**Maize plants**

*Growth traits*

In both seasons, samples of maize plants at 45 and 90 DAS were collected from each treatment to determine shoot height (cm), number of leaves/ plant, fresh weight ((FW) of plant (g), dry weight (DW) of plant (g).

*Estimation of photosynthetic pigments*

Chlorophyll a, chlorophyll b and carotenoids were extracted from the uppermost fourth fully expanded fresh leaf (0.1 g) of each plant at Vegetative (45 DAS) and late or flowering age (90 DAS) using 10 ml of N, N-dimethylformamide, following the procedure outlined by Moran [31]. The resulting extract was filtered and absorbance readings were recorded against a blank at wavelengths of 663.8, 646.8, and 470 nm using a JASCO V-750 spectrophotometer. The measured absorbance values were applied to calculate pigment concentrations which were expressed as mg g⁻¹ fresh weight of maize leaves.

$$\mathrm{Chl}\:\mathrm{a}\left(\mathrm{mg/g}\:\mathrm{FW}\right)=\left[12.64\times\mathrm{A}663.8-2.99\times\mathrm{A}646.8\right]\times\mathrm{W}\times1000\mathrm{V}$$

$$\mathrm{Chl}\:\mathrm{b}\left(\mathrm{mg/g}\:\mathrm{FW}\right)=\left[-5.6\times\mathrm{A}663.8+23.24\times\mathrm{A}646.8\right]\times\mathrm{W}\times1000\mathrm{V}$$

$$\mathrm{Total}\:\mathrm{Chlorophyll}=\mathrm{Chl}\:\mathrm{a}+\mathrm{Chl}\:\mathrm{b}$$

$$\mathrm{Carotenoids}\:\left(\mathrm{mg/g}\:\mathrm{FW}\right)=\left[1000\times\mathrm{A}470-1.28\times\mathrm{Chl}\:\mathrm{a}-56.7\times\mathrm{Chl}\:\mathrm{b}\right]230\times\mathrm{W}\times1000\mathrm{V}$$

*Chemical analysis of maize leaves*

The upper fourth leaf for each treatment at Vegetative (45 DAS) and late or flowering age (90 DAS) was collected, cleaned, dried and crushed to tiny powder using grinder and prepared for analysis.

*Total soluble sugar* (*TSS*)

The concentration of total soluble sugars in fixed maize oven dried leaf samples was assessed using the phenol–sulfuric acid colorimetric technique following the protocol originally developed by Dubois et al. [32]. Optical density was recorded at 490 nm using a JASCO V-750 spectrophotometer. Sugar content was calculated from a glucose calibration curve prepared against a reagent blank and expressed as mg g⁻¹ dry matter.

*Total phenolic compounds* (*TPC*)

Total phenolic compounds in maize leaf samples were quantified using the Folin–Ciocalteu colorimetric assay following the procedure described by Elzaawely and Tawata [33]. Absorbance readings were taken at 765 nm using a JASCO V-750 spectrophotometer. Phenolic content was estimated based on a calibration curve prepared with gallic acid and the results were expressed as mg gallic acid equivalents per gram of dry weight (mg GAE g⁻¹ DW).

#### Statistical analysis

Each recorded data parameter was explored for normal distribution according to the Shapiro-Wilk test (with a confidence level of *p* ≤ 0.05 for significant difference from a normal distribution). Most of the attributes were in normal or close to the normal distribution. Most of the attributes were in normal or close to the normal distribution. All recorded data from the applied experiments were subjected to combined analysis of variance (ANOVA) using CoStat software (version 6.303, 2004; Cohort Software), following the analytical framework described by Casella [34]. Mean comparisons were conducted using the Waller–Duncan multiple range test implemented through the MEANS option in the PROC GLM procedure [35].

## Results

### GC-MS fractionation of thyme oil terpenoids

GC–MS analysis of thyme essential oil (EO) resulted in the identification of fourteen volatile constituents, accounting for the majority of the total oil composition. The chromatographic represented in Table 3 profile was dominated by monoterpene compounds, with thymol being the principal constituent (31.79%). Other major components included γ-terpinene (14.76%) and p-cymene-related monoterpene hydrocarbons (12.09%), which together represented a substantial proportion of the oil. Oxygenated monoterpenes and sesquiterpenes were detected in lower amounts, contributing to the overall chemical diversity of the EO oil.

**Table 3:** Thyme oil terpenoids as fractionated by GC-MS

Peak	RT	Name	Formula	Area	Area Sum %
1	2.123	α-Pinene	C10H16	48,698,255	4.11
2	2.613	β-Pinene	C10H16	11,127,092	0.94
3	2.87	β-Myrcene	C10H16	15,808,109	1.34
4	2.98	α-Phellandrene	C10H16	32,812,629	2.77
5	3.153	P-Cymene [1,3-Cyclohexadiene, 1-methyl-4-(1-methylethyl)]	C10H16	143,133,988	12.09
6	3.295	Terpinene derivative [Cyclohexene, 1-methyl-4-(1-methylethylidene)]	C10H16	125,880,802	10.63
7	3.713	γ-Terpinene	C10H16	174,726,056	14.76
8	4.061	Terpinene derivative [Cyclohexene, 3-methyl-6-(1-methylethylidene)]	C10H16	53,414,469	4.51
9	5.277	Borneol derivatives [Bicyclo[3.1.0]hexan-2-ol, 2-methyl-5-(1-methylethyl)-, (1.alpha.,2.beta.,5.alpha]	C10H18O	105,233,294	8.89
10	5.438	α.-Terpineol	C10H18O	17,051,794	1.44
11	6.822	Oxygenated sesquiterpene [(1R,3E,7E,11R)-1,5,5,8-Tetramethyl-12-oxabicyclo[9.1.0]dodeca-3,7-diene]	C15H24O	51,118,133	4.32
12	7.144	Thymol	C10H14O	376,357,073	31.79
13	7.46	D-Verbenone	C10H14O	14,366,003	1.21
14	8.296	Caryophyllene	C15H24	14,013,969	1.18

### Characterization of formulated nanoemulsions

#### Foam test

The foam stability of the thyme oil nanoemulsions was significantly influenced by the concentration of the surfactant (Span 80) and the oil load (Table 4). Comparative analysis of the experimental runs reveals a non-linear relationship between surfactant concentration and foam retention. At a low surfactant concentration (1% Span 80, Run 1), foam stability was significantly lower (65%), likely due to an inadequate concentration of surfactant molecules to stabilize the air-liquid interface. Conversely, increasing the surfactant to 3% (Run 3) did not yield superior results; instead, stability decreased to 72%. This decline is attributed to reduced interfacial elasticity and increased liquid drainage often observed at higher surfactant saturation levels. The maximum stability was achieved in Run 2 (2% Span 80 and 2.5% thyme oil), which exhibited 90% foam retention after 30 min. This formulation significantly outperformed Run 4 (78%), where a lower oil concentration was used, and Run 5 (82%), which utilized a lower surfactant-to-oil ratio. These results validate that 2% Span 80 provides the optimal interfacial tension required to minimize foaming defects while maintaining the structural integrity of the NE-Th oil formulation. This low-foaming propensity is ideal for field-scale foliar applications, ensuring uniform coverage and preventing the loss of active ingredients during spraying.

**Table 4:** Foam test for nanoemulsion thyme oil (NE-Th oil)

Run	Span 80 (%)	Thyme Oil (%)	Cycles	Stability (%)
1	1	2	10	65
2	2	2.5	15	90
3	3	3	20	72
4	2	1	15	78
5	1.5	2.5	12	82

#### Evaluation of stability test results

**Fig. 1:**
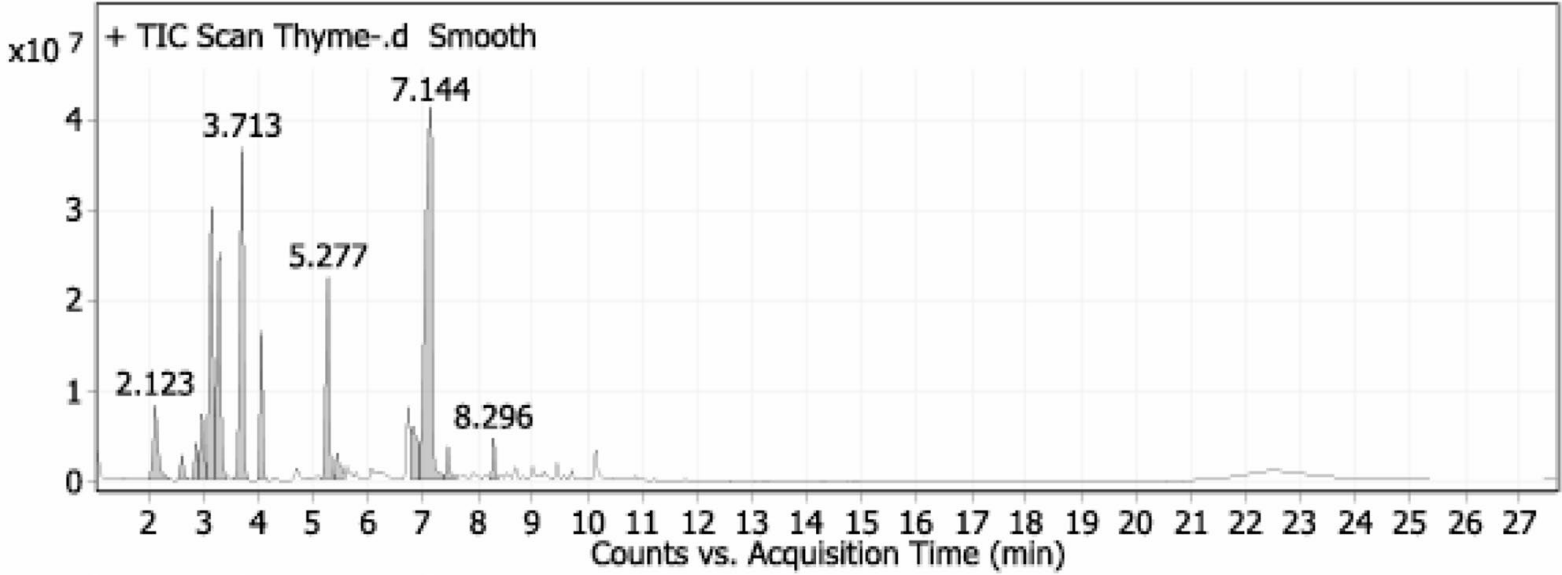
GC–MS chromatographic profiling of thyme essential oil

The stability of NE-Th oil and β-CD-Th oil formulations was evaluated under accelerated stress conditions to predict shelf-life and field performance (Table 5). The 2% and 3% Span-based nanoemulsions, as well as all encapsulated β-CD formulations (2%, 4%, and 6%), exhibited excellent kinetic stability, showing no phase separation even at 10,000 rpm. In contrast, the 1% Span nanoemulsion failed at the lowest speed (3,500 rpm), indicating that a minimum surfactant concentration is required to maintain the interfacial film against gravitational stress. The PDI (Polydispersity Index) served as a key indicator of droplet uniformity during thermal shock. The 2% Span NE maintained an ideal PDI < 0.3, whereas the 3% Span formulation showed a drastic increase to 1.2, suggesting significant droplet aggregation or Ostwald ripening during the freeze-thaw cycles. The encapsulated β-CD-Th oil demonstrated superior thermal resilience, maintaining a consistent PDI of 0.3 regardless of the oil concentration. Zeta potential values were used to assess the electrostatic stability of the particles. The β-CD-Th oil with 6% thyme oil exhibited the most robust stability profile, maintaining a constant Zeta potential of 22.13 mV across four heating-cooling cycles. This high surface charge contributes to a Grade A classification (ideal stability), as it provides sufficient repulsive forces to prevent particle clustering. While the nanoemulsions remained homogeneous, their lower Zeta potential values (up to -14 mV) (Fig. 1).

**Table 5:** Stability test (NE-Th oil & β-CD-Th oil)

Test	Specification	Nanoemulsion (span content )			Encapsulated (Thyme oil content)		
		1%	2%	3%	2	4	6
Centrifuge ( phase separation)	3500 rpm(30 min)	Yes	no	no	no	no	no
	7000 rpm(30 min)	yes	no	no	no	no	no
	10,000 rpm(30 min)	yes	no	no	no	no	no
Freeze thaw (PDI change)	-21°c	0.6	< 0.3	1.2	0.3	0.3	0.3
	10°c	0.6	< 0.3	1.2	0.3	0.3	0.3
	25°c	0.6	< 0.3	1.2	0.3	0.3	0.3
Heating-cooling (selection parameter zeta potential values)	1st cycle	-10	-14	-5	10	18	22.13
	2nd cycle	-10	-14	-5	10	18	22.13
	3rd cycle	-10	-14	-5	10	8	22.13
	4th cycle	-10	-14	-5	10	18	22.13
	5th cycle	-10	-14	-5	10	18	22.13
	6th cycle	-10	-14	-5	10	18	22.13
Creaming (observed by naked eye) storage at 25^o^C	24 hr.	B	A	A	A	A	A
	72 hr.	B	A	A	A	A	A
Creaming (observed by naked eye) storage at 40 ^o^C	7 days	C	A	B	C	B	A

#### Particle size and zeta potential of NE-Th oil and β-CD-Th oil

**Fig. 2:**
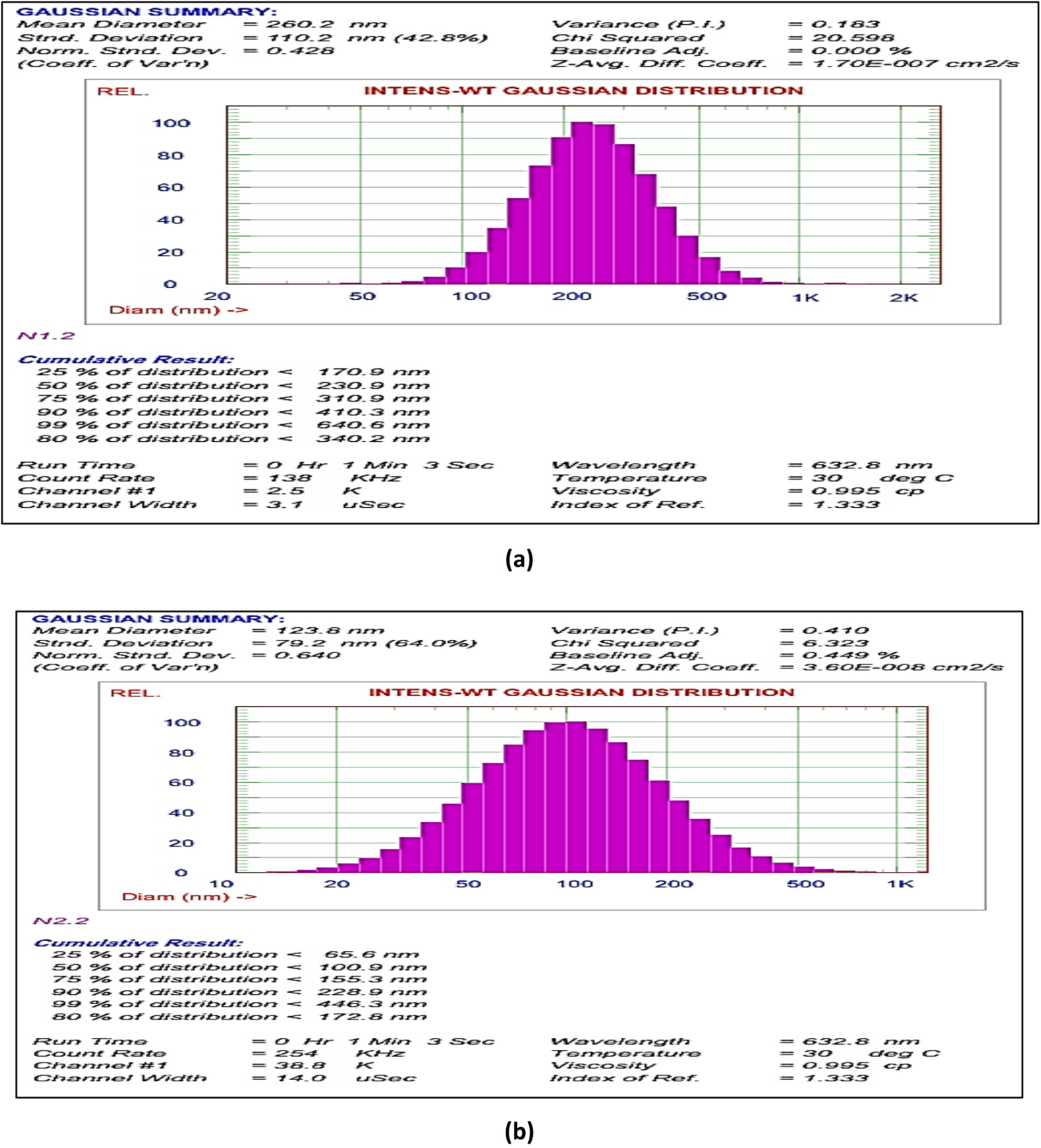
Particle size distribution of Th-NE (**a**) and β-CD-Th (**b**)

**Fig. 3:**
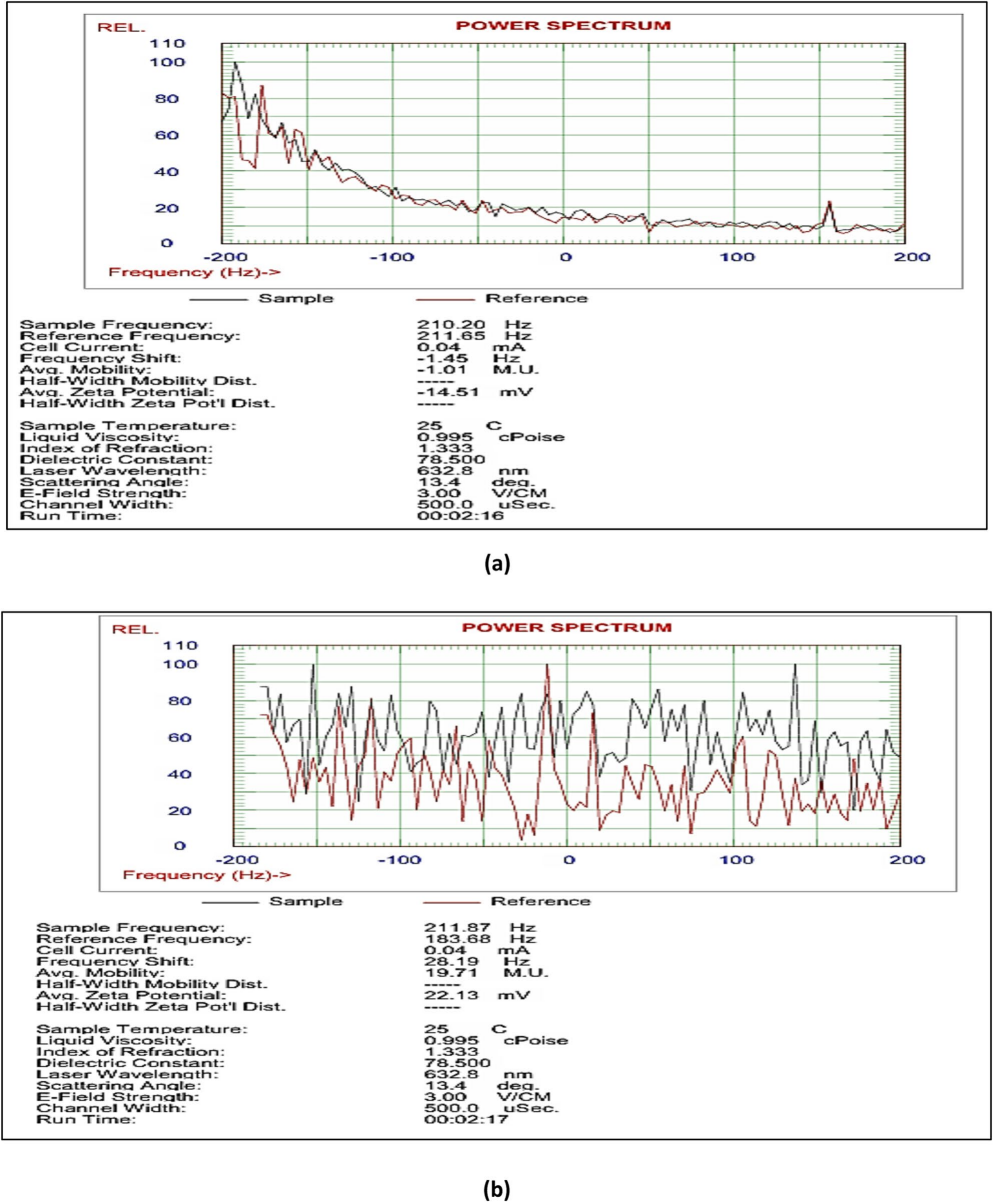
Zeta potential of Th-NE (**a**) and β-CD-Th (**b**)

The physical dimensions and surface charge of the nano-formulations were analyzed to evaluate their kinetic stability and potential for foliar uptake. As illustrated in Figs. 2 and 3, the two systems exhibited distinct physical profiles: Thyme Nanoemulsion (Th-NE): The mean droplet size was recorded at 260 nm with a PDI of 0.245. While slightly larger than the encapsulated particles, the PDI value confirms a narrow and uniform size distribution, which is critical for preventing Ostwald ripening. The Zeta potential was measured at -14.51 mV, suggesting that the stability of the emulsion is largely maintained through steric hindrance provided by the Span/Tween surfactant blend. β-CD Nanoencapsulation (β-CD-Th): This formulation exhibited a significantly smaller mean particle size of 123.8 nm and a superior PDI of 0.182. The lower PDI indicates a highly homogeneous population of inclusion complexes. Furthermore, the Zeta potential of + 22.13 mV indicates a higher degree of electrostatic repulsion compared to the nanoemulsion, contributing to the superior stability observed in the accelerated stress tests. The smaller particle size and lower PDI of the β-CD-Th formulation likely facilitate better penetration through the weed leaf cuticle, correlating with the high herbicidal efficacy observed in the preliminary trials. Suggest they rely more on steric stabilization from the Span 80 surfactant rather than pure electrostatic repulsion.

### A preliminary pot experiment

#### Weeds growth traits

Regarding fresh and dry biomass of both investigated weeds (barnyard grass and purslane), ANOVA exhibited that low concentrations of the applied thyme aqueous extract and thyme oil had significantly decreased weeds growth at 45 DAS as compared to mixed control (Table 6). It was noticed both weeds differed in their response to the investigated thyme extract. Thyme oil extract was more efficient in controlling both weeds than thyme leaves aqueous extract, especially at the high concentration. However, it was reported that thyme aqueous extract scored the highest suppression effect at the lowest concentration and this suppression decreased by increasing concentrations, scoring the highest reduction in weeds recorded at 10%, followed by 20% concentrations of both weeds at 20% concentration with no significant difference between them. However, thyme aqueous extract at 30% scored the lowest reduction in weeds biomasses with no significant difference from the unweeded control. In contrast, thyme oil reduced the biomass of both weeds and this reduction increased in parallel with concentration achieving the complete inhibition of both weeds at 9% concentration. The percentage reduction relative to the infected control was calculated to better clarify the herbicidal efficacy of the treatments. Thyme aqueous extract at 10 and 20% reduced barnyard grass dry weight by 59.24 and 57.86%, respectively, while reductions in purslane dry weight reached 95.09% and 54.72%. In contrast, thyme oil extract showed substantially stronger suppression. At 6% concentration, thyme EO oil reduced barnyard grass dry weight by 95.57% and purslane dry weight by 88.68%. These results demonstrate that thyme EO oil exhibited markedly higher herbicidal activity compared with the aqueous extract.

**Table 6:** Effect of thyme aqueous extract and thyme oil on fresh and dry weights of barnyard grass and purslane weeds at 45 DAS

Treatments		Barnyard grass		Purslane	
		FW (g)	DW (g)	FW (g)	DW (g)
Maize only (weed-free)		0.00 a	0.00 a	0.00	0.00
Control Maize + weeds (unweeded)		29.04 ± 0.48c	6.55 ± 1.48 cd	52.21 ± 1.37c	2.65 ± 1.16b
Thyme aqueous extract	10%	11.40 ± 0.74b	2.67 ± 0.47ab	5.34 ± 1.09a	0.13 ± 1.13a
	20%	11.80 ± 1.12b	2.76 ± 1.32ab	12.50 ± 1.15ab	1.20 ± 1.49a
	30%	26.43 ± 1.66c	8.63 ± 1.07d	44.32 ± 0.67bc	2.60 ± 0.56b
Thyme oil extract	3%	11.69 ± 1.04b	3.99 ± 1.32bc	6.64 ± 0.69a	0.48 ± 0.43a
	6%	1.01 ± 0.29a	0.29 ± 0.29a	1.52 ± 0.03a	0.30 ± 0.01a
	9%	0.00a	0.00a	0.00a	0.00a

#### Maize growth traits

Based on the data provided in Table (7), the recorded results indicated that the presence of weeds significantly suppressed maize growth. Hence, unweeded treatment showed the poorest performance across all growth parameters as compared to the weed-free control indicating the weed competition effect on maize plants. Weed free maize plants scored the highest shoot height 122.8 cm and dry biomass (13.2 g). This confirmed that uncontrolled weed biological stress significantly stunted maize development. Additionally, the application of thyme extracts generally mitigated this competition, allowing the maize to recover growth levels near those of the weed-free control. Maize treated with 20% thyme aqueous extract recorded stimulation in shoot height (122.2 cm) and dry weight biomass (12.2 g), making it statistically identical to the weed-infested control. While thyme EO oil 6 and 3% concentrations came in the second rank scoring 113.3 and 108.3 cm maize shoot Hight and 11.9 and 11.5 g dry weights of maize plants, respectively as compared to unweeded control. Although 9% thyme oil scored complete suppression for weeds but maize plants showed lower stimulatory response in shoot height (96.3 cm) and dry weight (11.4 g), but still higher than unweeded infested control treatment.

**Table 7:** Effect of thyme aqueous extract and thyme oil on maize growth parameters at 45 DAS

Treatments			Shoot height	No. of leaves	FW plant^− 1^ (g)	DW plant^− 1^ (g)
Control	Maize only (weed-free)		122.8 ± 1.68a	7.2 ± 0.19a	129.06 ± 1.52a	13.2 ± 0.62a
	Maize+ weeds (unweeded)		90.3 ± 2.52d	6.3 ± 0.58b	50.70 ± 2.04c	4.6 ± 0.32c
Thyme aqueous extract		10%	116.8 ± 2.14ab	7.0 ± 0.33ab	94.35 ± 1.36b	9.9 ± 0.93b
		20%	122.2 ± 2.83a	7.2 ± 0.40a	126.88 ± 1.55a	12.2 ± 0.15a
		30%	105.3 ± 1.53bc	6.9 ± 0.35ab	89.83 ± 1.32b	11.1 ± 0.23ab
Thyme oil extract		3%	108.3 ± 1.67bc	7.1 ± 0.19a	95.42 ± 2.98b	11.5 ± 2.42ab
		6%	113.3 ± 1.76ab	7.1 ± 0.17a	105.26 ± 2.00b	11.9 ± 0.43ab
		9%	96.3 ± 1.53 cd	6.3 ± 0.58b	93.17 ± 1.67b	11.4 ± 1.23ab

### The main pot experiment

#### Efficiency in controlling weeds

**Fig. 4:**
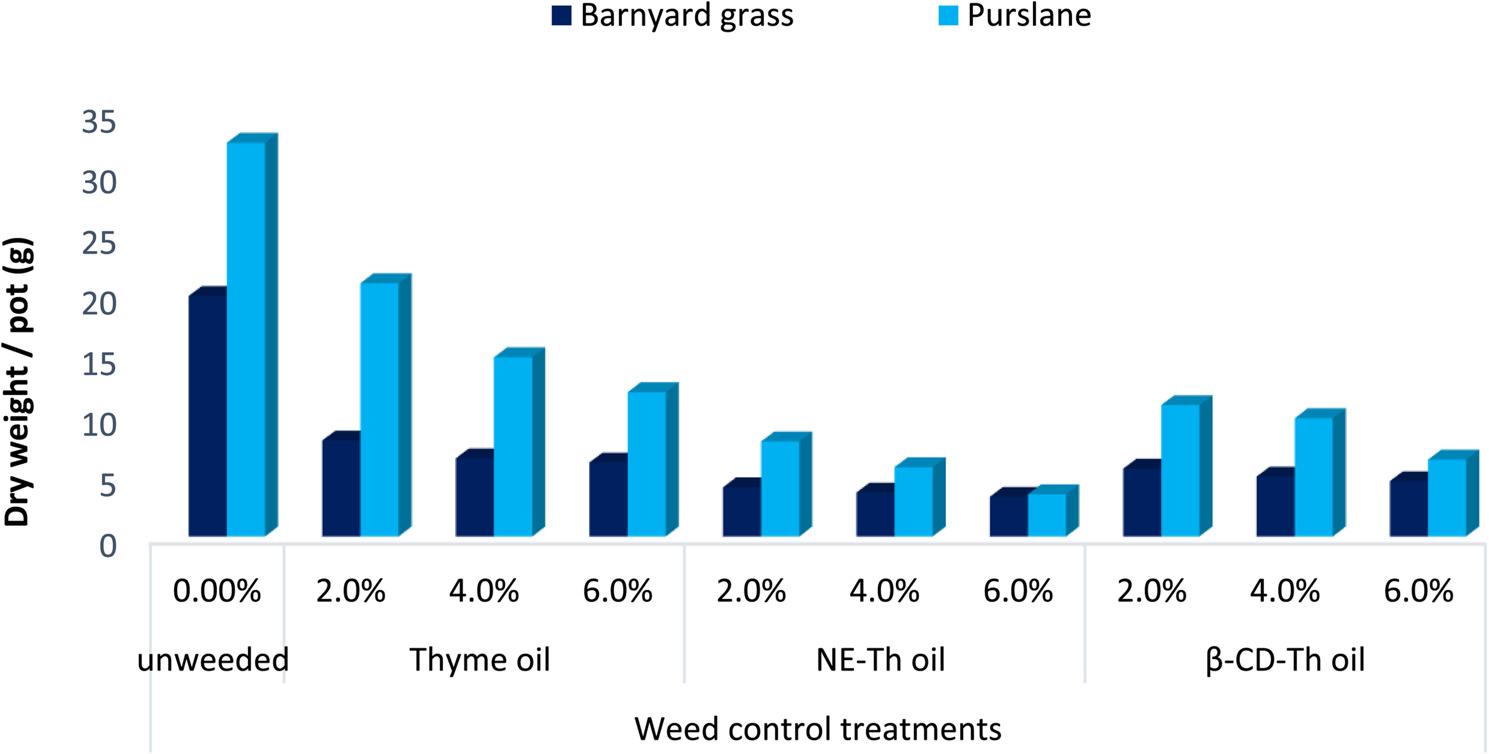
Effect of thyme oil, its nanoemulsion and β-cyclodextrin nanoencapsulation on dry weight of weeds at 90 DAS (Values are means ± SE (*n* = 3)).; SE = 0.17 for Barnyard grass and 0.36 for Purslane

As presented in Tables 8 and 9, thyme EO, its nanoemulsion (NE-Th oil) and β-cyclodextrin–encapsulation (β-CD-Th oil) applied at successive concentrations significantly reduced the fresh and dry biomasses of barnyard grass and purslane weeds at 45 and 90 DAS. Recorded results presented that each weed’s rate of reduction was directly attributed with increasing of the thyme oil and its two different nano phases concentrations. At both sampling times, NE-Th and β-CD-Th oil treatments were more effective in suppressing weed growth than the unmodified crude thyme oil (Fig. 4). Moreover, the two weed species exhibited different responses to the applied treatments and sampling time. Accordingly, at 45 DAS, the lowest barnyard grass biomass was recorded under NE-Th oil at 6 and 4%, followed by β-CD-Th oil at 6 and 4%, compared with the unweeded control. These highest suppressive treatments achieved barnyard grass reduction dry weight reached to 84.94, 80.71, 79.53 and 73.41%, respectively. At 90 DAS as tabulated in Table 9, barnyard grass biomass was most effectively reduced by NE-Th oil at 6, 4 and 2%, followed by β-CD-Th oil at the same concentrations 83.48, 81.72, 79.55, 76.08, 75.08 and 71.80%, successively. Regarding purslane, NE-Th oil applied at 6, 4, and 2% showed the highest herbicidal efficacy at 45 DAS causing reduction reached to 92.03, 88.84, 83.60%, respectively. However, at 90 DAS, NE-Th oil at 6 and 4% remained the most effective treatments, followed by β-CD-Th oil at 6% achieving reduction equal 89.27, 82.41, 80.47%, successively, relative to the unweeded control.

**Table 8:** Effect of thyme oil, its nanoemulsion and β-cyclodextrin nanoencapsulation on fresh and dry weight of weeds at 45 DAS (the result represent mean of two seasons)

Treatments			At 45 DAS			
			Barnyard grass		Purslane	
			FW (g)	DW (g)	FW (g)	DW (g)
Control	Maize only (weed-free)		0.00	0.00	0.00	0.00
	Maize+ Weeds (unweeded)		8.83 ± 0.75d	4.25 ± 0.20 h	96.66 ± 1.70 g	21.96 ± 0.43 g
Thyme oil		2.0%	5.33 ± 0.12c	2.52 ± 0.08 g	49.68 ± 2.17f	11.40 ± 0.52f
		4.0%	4.91 ± 0.91c	2.17 ± 0.18f	31.83 ± 2.27e	6.99 ± 0.29d
		6.0%	3.02 ± 0.71ba	1.44 ± 0.26ed	17.15 ± 1.53c	4.11 ± 0.36b
NE-Th oil		2.0%	2.96 ± 0.08ba	1.36d ± 0.31c	13.63 ± 0.79cb	3.60 ± 0.21b
		4.0%	2.35 ± 0.03ba	0.82 ± 0.05a	11.57 ± 0.87b	2.45 ± 0.38a
		6.0%	1.94 ± 0.38a	0.64 ± 0.05a	5.26 ± 0.99a	1.75 ± 0.21a
β-CD-Th oil		2.0%	3.54 ± 0.42b	1.64 ± 0.15d	35.55 ± 0.95e	7.96 ± 0.44e
		4.0%	2.82 ± 0.18ba	1.13 ± 0.07cb	22.04 ± 1.97d	5.11 ± 0.69c
		6.0%	2.49 ± 0.03ba	0.87 ± 0.07ba	16.49 ± 0.50c	3.63 ± 0.48b

**Table 9:** Effect of thyme oil, its nanoemulsion and β-cyclodextrin nanoencapsulation on fresh and dry weight of weeds at 90 DAS (the result represent mean of two seasons)

Treatments			At 90 DAS			
			Barnyard grass		Purslane	
			FW (g)	DW (g)	FW (g)	DW (g)
Control	Maize only (weed-free)		0.00	0.00	0.00	0.00
	Maize+ Weeds (unweeded)		40.04 ± 0.55 g	19.86 ± 0.8 g	156.27 ± 2.82 h	32.52 ± 0.91 h
Thyme oil		2.0%	17.33 ± 0.29f	7.93 ± 0.89f	83.56 ± 1.55 g	20.92 ± 0.77 g
		4.0%	13.97 ± 0.26e	6.45 ± 0.17e	60.81 ± 2.42f	14.80 ± 0.45f
		6.0%	12.20 ± 0.61d	6.11 ± 0.18e	54.02f ± 1.73e	11.91 ± 0.33e
NE-Th oil		2.0%	8.32 ± 0.18b	4.06 ± 0.10b	29.73 ± 1.00cb	7.85 ± 0.44c
		4.0%	7.29 ± 0.04a	3.63 ± 0.05ba	23.70 ± 2.78b	5.72 ± 0.60b
		6.0%	6.52 ± 0.04a	3.28 ± 0.05a	12.25 ± 1.00a	3.49 ± 0.44a
β-CD-Th oil		2.0%	11.39 ± 0.20d	5.60 ± 0.16a	47.64 ± 1.50ed	10.85 ± 0.98e
		4.0%	10.43 ± 0.14c	4.95 ± 0.10c	37.41 ± 2.94dc	9.77 ± 0.30d
		6.0%	9.55 ± 0.25c	4.57 ± 0.06c	25.33 ± 1.61b	6.35 ± 0.37b

#### Maize growth parameters

As shown in Tables 10 and 11, the natural bioherbicide thyme EO, along with its nanoemulsion and β-CD-Th oil nanocomplex, significantly enhanced maize growth parameters at both growth ages, including shoot height, number of leaves per plant, fresh weight, and dry weight, compared with the unweeded control. Notably, both modified formulations of thyme EO i.e. nanoemulsion and nanoencapsulation exhibited greater stimulatory effects on maize growth than the crude thyme oil. At 45 DAS, weed-free healthy maize plants came in the first rank in stimulation of the aforementioned growth parameters. β-CD-Th oil at 4, 6% and NE-Th oil at the same concentrations, respectively, came in the second rank after healthy plants with no significant difference between them in most recorded parameters. These superior treatments induced maize dry weight from11.66 g dry biomass of unweeded control to (20.90, 20.18, 19.76 and 18.64 g, respectively). However, at 90 DAS, NE-Th oil showed superior performance over the β-CD-encapsulated formulation in promoting maize growth (Table 8). NE-Th oil at 6% ranked first, followed by β-CD-Th oil at 4% and NE-Th oil at 4 and 2% with no significant difference between them. These enhancer treatments increased maize plant dry weight over unweeded control treatment dry biomass (24.37 g) to 50.83, 45.48, 44.44 and 41.32 g, respectively. A good noticed achievement was that these superior treatments proceeded on healthy weed-free maize plants that ranked them in stimulation of maize plants with significant difference in fresh and dry biomass of maize plants.

**Table 10:** Effect of thyme oil, its nanoemulsion and β-cyclodextrin nanoencapsulation on maize growth parameters at 45 DAS (the results represent mean of two seasons)

Treatments			Shoot height (cm)	No. of leaves plant^− 1^	FW plant^− 1^ (g)	DW plant^− 1^ (g)
Control	Maize only (weed-free)		129.5 ± 4.0a	7.8 ± 0.4a	91.51 ± 2.68a	25.85 ± 2.86a
	Maize+ Weeds (unweeded)		98.3 ± 2.1c	7.1 ± 0.2a	35.28 ± 1.86d	11.66 ± 1.66e
Thyme oil		2.0%	103.0 ± 2.6bc	7.1 ± 0.0a	37.94 ± 2.29d	13.81 ± 0.42 cd
		4.0%	105.7 ± 4.4bc	7.2 ± 0.5a	42.05 ± 1.76 cd	14.20 ± 0.52 cd
		6.0%	100.6 ± 4.7bc	7.1 ± 0.2a	37.73 ± 1.37d	12.33 ± 1.75de
NE-Th oil		2.0%	106.2 ± 4.2bc	7.2 ± 0.7a	49.72 ± 3.81bc	16.64 ± 2.63bc
		4.0%	116.1 ± 3.3ab	7.6 ± 0.4a	62.05 ± 3.91bc	19.76 ± 0.68b
		6.0%	113.1 ± 4.2ab	7.6 ± 0.5a	57.70 ± 2.81bc	18.64 ± 0.96bc
β-CD-Th oil		2.0%	111.7 ± 4.2ab	7.2 ± 0.7a	50.16 ± 2.57bc	17.6 ± 1.39bc
		4.0%	118.7 ± 2.5ab	7.6 ± 0.4a	85.83 ± 2.58a	20.90 ± 1.67b
		6.0%	116.3 ± 2.6ab	7.3 ± 0.0a	70.33 ± 3.13ab	20.18 ± 1.52b

**Table 11:** Effect of thyme oil, its nanoemulsion and β-cyclodextrin nanoencapsulation on maize growth parameters at 90 DAS (the results represent mean of two seasons)

Treatments			Shoot height (cm)	No. of leaves plant^− 1^	FW plant^− 1^ (g)	DW plant^− 1^ (g)
Control	Maize only (weed-free)		162.7 ± 2.1ab	13.5 ± 0.5a	122.69 ± 3.65bc	40.09 ± 0.68bc
	Maize+ Weeds (unweeded)		148.5 ± 3.3d	10.3 ± 0.8b	72.65 ± 3.25e	24.37 ± 0.99e
Thyme oil		2.0%	150.3 ± 1.8d	12.0 ± 0.0ab	82.67 ± 2.52de	26.71 ± 1.77de
		4.0%	154.0 ± 2.0 cd	12.2 ± 0.3ab	103.90 ± 2.44cb	37.12 ± 0.40bc
		6.0%	157.3 ± 2.5bc	12.5 ± 0.5ab	117.67 ± 2.41bc	38.85 ± 1.33bc
NE-Th oil		2.0%	166.7 ± 2.0ab	13.7 ± 0.6a	131.84 ± 2.19ab	41.32 ± 1.17ab
		4.0%	171.2 ± 3.6ab	13.7 ± 0.5a	134.50 ± 2.19ab	44.44 ± 1.29ab
		6.0%	179.8 ± 2.3a	14.3 ± 0.3a	167.80 ± 2.14a	50.83 ± 0.69a
β-CD-Th oil		2.0%	156.0 ± 2.0bc	12.3 ± 0.6ab	106.05 ± 1.84bc	35.48 ± 0.73bc
		4.0%	174.5 ± 2.8ab	13.8 ± 0.3a	143.20 ± 2.98ab	45.48 ± 0.69ab
		6.0%	153.0 ± 2.6 cd	12.0 ± 0.0ab	98.60 ± 3.15 cd	33.95 ± 1.54 cd

##### Photosynthetic pigment contents

Table 12 presents the changes in photosynthetic pigments (chlorophyll a, chlorophyll b, and carotenoids, µg/g FW) in maize leaves at 45 and 90 DAS as affected by thyme EO oil and its nano-formulations. The unweeded treatment markedly reduced total photosynthetic pigments to 0.493 and 4.028 µg/g FW at 45 and 90 DAS, respectively, compared with the weed-free control (1.510 and 6.260 µg/g FW). At 45 DAS, the highest total pigment content was recorded in the weed-free control (1.510 µg/g FW), followed by β-CD-Th oil at 4% (1.151 µg/g FW) and NE-Th oil at 4% (1.114 µg/g FW). At 90 DAS, nano-formulations outperformed the control, where NE-Th oil at 2% recorded the highest value (8.873 µg/g FW), representing an increase of approximately 41.7% over the weed-free control, followed by β-CD-Th oil at 4% (7.271 µg/g FW).Increasing the concentration to 6% reduced pigment contents (e.g., 5.831 µg/g FW for NE-Th oil and 6.011 µg/g FW for β-CD-Th oil at 90 DAS), although these values remained higher than the unweeded treatment.

**Table 12:** Effect of thyme oil, its nanoemulsion and β-cyclodextrin nanoencapsulation on photosynthetic pigment contents at 45 and 90 DAS (the results represent mean of two seasons)

Treatments		At 45 DAS				At 90 DAS			
		Chla	Chlb	Carotenoids	Total Chlorophyll	Chla	Chlb	Carotenoids	Total Chlorophyll
Control	Maize only (weed-free)	1.179 ± 0.131a	0.109 ± 0.022a	0.222 ± 0.191a	1.510 ± 0.323a	4.351 ± 0.705bc	0.969ab	0.940 ± 0.386ab	6.260c
	Maize+ Weeds (unweeded)	0.343 ± 0.118c	0.024 ± 0.012c	0.125 ± 0.042a	0.493 ± 0.171c	2.928 ± 0.329f	0.701 ± 0.028b	0.399 ± 0.201c	4.028 ± 0.157f
Thyme oil	2.0%	0.432 ± 0.069c	0.0290 ± 012bc	0.140 ± 0.038a	0.602 ± 0.115bc	3.384 ± 0.146ef	0.982 ± 0.107ab	0.808 ± 0.011bc	5.174 ± 0.435de
	4.0%	0.424 ± 0.058c	0.0380 ± 016bc	0.131 ± 0.018a	0.592 ± 0.062c	3.750 ± 0.262de	0.771 ± 0.047b	0.848 ± 0.066bc	5.369 ± 0.369de
	6.0%	0.396 ± 0.006c	0.030 ± 0.001bc	0.141 ± 0.001a	0.567 ± 0.006c	3.309 ± 0.669ef	0.723 ± 0.036b	0.805 ± 0.038bc	4.837 ± 0.157e
NE-Th oil	2.0%	0.452 ± 0.041c	0.036 ± 0.027bc	0.159 ± 0.133a	0.647 ± 0.176bc	5.768 ± 0.236a	1.677 ± 0.451a	1.428 ± 0.264a	8.873 ± 0.547a
	4.0%	0.818 ± 0.303b	0.071 ± 0.039b	0.225 ± 0.070a	1.114 ± 0.401ab	4.844 ± 0.481bc	1.1030 ± 0.040ab	1.032 ± 0.408ab	6.979 ± 0.410bc
	6.0%	0.526 ± 0.235c	0.037 ± 0.018bc	0.178 ± 0.137a	0.777 ± 0.325b	4.073 ± 1.045 cd	0.895 ± 0.073b	0.863 ± 0.640bc	5.831 ± 0.693 cd
β-CD-Th oil	2.0%	0.538 ± 0.081c	0.039 ± 0.033bc	0.170 ± 0.074a	0.747 ± 0.163bc	3.749 ± 0.315de	0.831 ± 0.560b	0.842 ± 0.081bc	5.422 ± 0.221de
	4.0%	0.824 ± 0.122b	0.052 ± 0.012bc	0.275 ± 0.036a	1.151 ± 0.169a	5.015 ± 0.403ab	1.171 ± 0.688ab	1.085 ± 0.100ab	7.271 ± 0.533ab
	6.0%	0.806 ± 0.195b	0.044 ± 0.007bc	0.268 ± 0.075a	1.118 ± 0.274ab	4.237 ± 0.206bc	0.825 ± 0.679b	0.949 ± 0.164ab	6.011 ± 0.997c

##### Total soluble sugar (TSS) and total phenolic content (TPC) in maize leaves

Table (13) showed the biochemical response of maize leaves to total Soluble Sugars (TSS) and total Phenolic compounds (TPC) at 45 and 90 DAS. TSS and TPC were significantly influenced by weed competition and the subsequent application of thyme-based treatments. TSS levels are a primary indicator of photosynthetic efficiency and carbon assimilation. The control (Maize + Weeds) showed the lowest TSS accumulation at both 45 DAS (21.72 mg/g DW) and 90 DAS (35.97 mg/g DW), indicating severe metabolic suppression due to weed competition. At 45 DAS, β-CD-Th oil at 4.0% reached the highest TSS level (46.84 mg/g DW), significantly outperforming the unweeded control. At 90 DAS, the NE-Th oil at 6.0% recorded the highest TSS value (85.30 mg/g DW), followed closely by the 4.0% NE-Th oil concentration (82.84 mg/g DW). This indicates that the nanoemulsion provides a more sustained metabolic boost as the plant matures.

**Table 13:** Effect of thyme oil, its nanoemulsion and β-cyclodextrin nanoencapsulation on total soluble sugar (TSS) and total phenolic content (TPC) in maize leaves at 45 and 90 DAS (mean of two seasons)

Treatments			45 DAS		90 DAS	
			TSS	TPC	TSS	TPC
Control	Maize only (weed-free)		36.62 ± 2.00bc	1.28 ± 0.02a	69.13 ± 0.12d	1.64 ± 0.20ab
	Maize+ Weeds (unweeded)		21.72 ± 0.20e	0.88 ± 0.01d	35.97 ± 3.01i	1.15 ± 0.05d
Thyme oil		2.0%	23.48 ± 0.21e	1.00 ± 0.01c	52.93 ± 1.57 h	1.18 ± 0.02d
		4.0%	25.55 ± 0.16de	1.09 ± 0.00bc	59.20 ± 0.50 g	1.27 ± 0.02 cd
		6.0%	27.27 ± 3.00de	1.09 ± 0.01bc	64.36 ± 1.52e	1.43 ± 0.02bc
NE-Th oil		2.0%	28.05 ± 0.60de	1.11 ± 0.00b	69.40 ± 0.12d	1.64 ± 0.05ab
		4.0%	32.53 ± 0.31 cd	1.23 ± 0.06a	82.84 ± 0.02b	1.76 ± 0.36ab
		6.0%	39.95 ± 11.91ab	1.24 ± 0.03a	85.30 ± 0.89a	1.90 ± 0.51a
β-CD-Th oil		2.0%	32.33 ± 0.52 cd	1.13 ± 0.01b	62.11 ± 0.06f	1.46 ± 0.01bc
		4.0%	46.84 ± 46.84a	1.30 ± 0.02a	72.11 ± 1.46c	1.50 ± 0.01bc
		6.0%	43.92 ± 1.11a	1.24 ± 0.17a	54.57 ± 0.80 h	1.50 ± 0.02bc

TPC reflect the plant’s secondary metabolism and its ability to defend against environmental stressors. Raw thyme oil treatments showed significantly decreased TPC values than unweeded control (ranging from1.00 to 1.09 mg/g DW at 45 DAS 1.18 to 1.43 mg/g DW at 90 DAS). This confirms that raw oil is less effective at stimulating the plant’s protective secondary metabolism. The use of nanoformulations generally restored or exceeded the TPC levels of the uninfected control. At 45 DAS, β-CD-Th oil (4%) scored the highest level of TPC (1.30 mg/g DW) as compared with control. At 90 DAS, 6.0% NE-Th oil produced the highest TPC concentration (1.90 mg/g DW) as compared to corresponding unweeded control.

## Discussion

Essential oils (EOs), particularly those from *Thymus* species, have been increasingly investigated as natural herbicides due to their allelopathic potential and high biodegradability compared with synthetic chemicals [8]. In accordance with our GC-MS fractionation, terpenoids are the most abundant constituents in thyme EO such as thymol, carvacrol, p-cymene and γ-terpinene exert inhibitory effects on weed germination and growth through involvement in fundamental plant metabolic processes, including photosynthesis, respiration, and hormonal regulation of growth and development [36]. Most terpenes primarily act in ecological contexts, where they influence the metabolism of surrounding organisms either positively or negatively [37]. In more details, the lipophilic nature of these oily compounds promotes their accumulation within cellular membranes, where EOs can induce membrane destabilization by increasing fluidity or inhibiting membrane enzymes. This disruption caused by monoterpenes is likely responsible for their herbicidal activity and subsequent cell death [38]. Additionally, Verdeguer et al. [39] explained the selective response of weeds or economical crops to metabolites attributed to species-specific responses that likely reflect differences in cuticle permeability and inherent physiological resilience among weeds. Although lacking specific studies isolating these terpenoids within thyme oil and investigating its herbicidal mode of action, their presence in thyme EO profiles may support the possibility of allelopathic action of monoterpenes by enhancing cuticular penetration and phytotoxic effects [36]. This may explain our findings that thyme EO effectively suppressed barnyard grass and purslane which correlates with this broader evidence that thyme oil can significantly reduce weed establishment.

Despite our observed superior performance of the unformulated thyme EO bioactivity in controlling weeds [8], their direct agricultural application faces challenges including volatility, low water solubility and rapid environmental degradation [9]. Formulating EOs into nanoemulsions has emerged as a promising strategy to overcome these limitations by enhancing dispersibility, stability, and delivery efficiency in foliar applications [10]. Nanoemulsions markedly reduce droplet size, increase surface area and improve wettability, all of which can enhance interactions with plant tissues and herbicidal efficacy at lower EO concentrations compared with coarse emulsions [11].

Nanoemulsions of fennel (*Foeniculum vulgare* Mill.) EO produced via ultrasonic emulsification have been reported to exhibit pronounced bioherbicidal activity against several wheat-associated weeds, including *Phalaris minor*, *Avena ludoviciana*, *Rumex dentatus*, and *Medicago denticulata*. These nanoemulsions showed greater efficacy than conventional EO emulsions, markedly suppressing seed germination and seedling growth. Their enhanced activity was associated with increased membrane permeability and electrolyte leakage, indicating membrane disruption. Elevated activities of antioxidant enzymes such as catalase and peroxidase further suggest that nanoemulsion treatments induced oxidative stress through excessive generation of reactive oxygen species. Moreover, fennel oil nanoemulsions demonstrated high physicochemical stability, maintaining integrity under centrifugation, dilution, and storage at ambient temperature for up to 30 days [40]. Comparable outcomes were observed in *Amaranthus retroflexus* and *Chenopodium album* treated with nanoemulsions of *Satureja hortensis* EO formulated using a low-energy catastrophic phase inversion technique. Under controlled laboratory conditions, increasing nanoemulsion concentrations progressively reduced germination percentage, germination speed index, and both root and shoot elongation of the tested weeds. In greenhouse experiments, foliar application at the 2–4 true-leaf age resulted in higher mortality rates and significant reductions in fresh and dry biomass, leaf area, and the lengths of primary roots and shoots. A dose-dependent decline in total chlorophyll content was also recorded, accompanied by loss of membrane integrity and increased electrolyte leakage. These nanoemulsions exhibited narrow droplet size distribution, droplet diameters below 130 nm, and good storage stability over 30 days at room temperature [41]. Collectively, these findings highlight nanoemulsion-based formulations as an effective strategy to address the inherent limitations of EOs, particularly their low water solubility and high volatility. By enhancing stability, bioavailability, and biological activity, nanoemulsions represent a promising and environmentally sustainable approach for improving the herbicidal potential of EOs and supporting weed management in organic and low-input agricultural systems.

In addition to nanoemulsification, encapsulating EOs in β-cyclodextrin inclusion complexes (βc-thyme oil) can further enhance their agronomic utility by reducing volatility, extending shelf life, and enabling gradual release of active volatiles under environmental conditions [42]. Cyclodextrins (CDs) are cyclic oligosaccharides with a hydrophilic exterior and a lipophilic cavity that can encapsulate hydrophobic molecules like monoterpenoids, improving aqueous solubility and stability of the guest compounds [17]. While most research on β-cyclodextrin–EO complexes has focused on antimicrobial and pharmaceutical applications, similar stabilization mechanisms are applicable to agrochemical use, where extended release and protection against degradation can increase efficacy at lower doses [43, 44]. Encapsulation of EOs using CDs is recognized as an effective approach for enhancing their physicochemical characteristics, including solubility and stability [45]. Complexation with CDs can transform liquid EOs into water-dispersible solid powders, which facilitates handling and application. Numerous studies have confirmed the advantages of EO encapsulation, demonstrating that cyclodextrin inclusion can function as a stable delivery system for Mexican oregano EO and its individual components [46, 47]. In particular, β-cyclodextrin (β-CD) encapsulation has been shown to improve the antimicrobial efficacy of key phenolic constituents such as carvacrol [48, 49] and thymol [50]. Consistent with these findings, Arana-Sánchez et al. [51] demonstrated that β-cyclodextrin microencapsulation preserved the antioxidant and antimicrobial properties of *Lippia graveolens* EO and highlighted that the chemical composition of the oil plays a critical role in determining encapsulation efficiency. Although β-Cs inclusion research specific to thyme EO herbicidal action remains limited, analogous benefits can be inferred: improved handling, reduced loss of volatile constituents, and enhanced delivery under greenhouse conditions could contribute to the higher weed suppression and maize growth stimulation observed in our study.

A foam test was conducted to assess the stability of the formulated product, while a stability test was performed on Span-stabilized thyme systems. This emphasized the importance of thermodynamic stress protocols to maintain long-term integrity within the prepared matrix, distinguishing between different metastable formulations and truly stable ones through multicycle challenges. The absence of creaming confirms the lipophilic efficacy of Span in preventing coalescence, which is particularly suitable for polymer matrices. Conversely, visible creaming (grades B and C) at low surfactant concentrations (< 1%) in the nanoemulsion or lower oil content (grades B and C) indicates the occurrence of Ostwald ripening, which can be optimized through ultrasonication (10 min) or the use of Tween as a co-surfactant. These findings correlate with dynamic light scattering (DLS) results (polydispersity index < 0.3), aligning with the outcomes of freeze-thaw tests. The nanoemulsion formulated with 2% emulsifiers demonstrated enhanced stability, and notably, no creaming was observed after a storage period of 7 days. Similar results were noted for encapsulated nanoemulsion samples that contained 6% oil content. The creaming stability results were well correlated with zeta potential measurements, as neither sedimentation nor creaming was observed when the zeta potential value exceeded − 14 mV for nanoemulsions and 22.13 for encapsulated nanoemulsions.

Employing a centrifugal technique to assess phase separation as an indicator for stability testing, we observe that there is no phase separation following centrifugation at various RPMs for 30 min, with an additional hold for 10 min, indicating good stability. In the evaluation of heating and cooling for both the prepared nanoemulsion and the encapsulated nanoemulsion, the zeta potential values remain unchanged even after six cycles. The ideal PDI values for the freeze-thaw test were observed in the 2% surfactant prepared nanoemulsions and in the 2, 4 and 6% thyme oil content for the encapsulated prepared nanoemulsion. A high zeta potential value theoretically indicates high stability, whereas a low zeta potential value suggests low stability, as a low repulsive force between droplets prevents flocculation and close contact [52]. The findings of this study may be significantly attributed to inadequate repulsive forces surrounding the oil droplets, which are essential for electrostatic stabilization. This phenomenon is facilitated by emulsifiers that inhibit droplet coalescence and promote their combination [53]. Consequently, the gravitational force acting on the various aqueous phases and the density of the oil may lead to coalescence, resulting in an increase in particle size that could further destabilize and lead to sedimentation [54].

The degree of electrical repulsion force among particles was evaluated using Zeta potential, which was utilized to determine the stability of the nanoemulsions. The Zeta potential values for three nanoemulsion samples, differing in their emulsifier concentrations of 1, 2 and 3%, were recorded as (− 10, − 14, and − 5), respectively. A high Zeta potential value theoretically indicates high stability, whereas a low Zeta potential value signifies low stability, as a low repulsive force between droplets allows for flocculation and close contact [52]. To prevent coalescence, the electrostatic repulsion among droplets in a nanoemulsion should be maintained at a Zeta potential value of ± 30 mV [55]. The findings of this study indicated that emulsifier concentrations influenced the stability of the NSO nanoemulsions. Consequently, the A10 sample demonstrated the highest Zeta potential value, reflecting the strongest electrostatic repulsion force between droplets, which resulted in a stable emulsion. In contrast, the 1 and 3% samples did not meet the minimum Zeta potential requirement, respectively, indicating low stability of the nanoemulsion [52].

Moreover, the stimulation of maize growth parameters seen with nanoformulated and inclusion-complexed thyme EO treatments may be due both to selective phytotoxicity against weeds (reducing competitive stress for the crop) and to low-dose harmful effects. As concentration dependent, low concentrations of plant secondary metabolites can induce stress responses or activate beneficial metabolic pathways in crops, enhancing root and shoot development, photosynthesis rates, or antioxidant defense systems [56]. Additionally, the contents of carotenoid and total chlorophyll were dose-dependent on the investigated treatments. The mechanism that leads to the reduction of photosynthetic pigments by EOs is mostly unknown. However, it has been suggested that the ability of molecules in these pigments to react with reactive oxygen can be a potential reason for decreasing pigments at times of stress [57]. This consequently affected total soluble sugar and total phenolic content [12].

## Conclusion

Both nanoemulsion and β-cyclodextrin inclusion complexes improved the stability, delivery, and herbicidal efficacy of thyme essential oil, with the 4–6% nanoemulsion formulation achieving the highest weed control and maize growth. These findings highlight a practical, eco-friendly strategy for sustainable weed management, reducing reliance on synthetic herbicides while maintaining crop productivity. 

## Data Availability

The row data are available on request.

## Abbreviations

EO: Essential oil
β-CD-Thyme oil: β-cyclodextrin thyme oil
NE-Th oil: Nanoemulsion thyme oil
DAS: Days after sowing
chla: Chlrophyll
chlb: Chlorophyllb
TSS: Total soluble sugar
TPC: Total phenolic content
